# Exploring changes in depression and radiology-related publications research focus: A bibliometrics and content analysis based on natural language processing

**DOI:** 10.3389/fpsyt.2022.978763

**Published:** 2022-11-30

**Authors:** Kangtao Wang, Fengbo Tan, Zhiming Zhu, Lingyu Kong

**Affiliations:** ^1^Department of General Surgery, Xiangya Hospital, Central South University, Changsha, Hunan, China; ^2^National Clinical Research Center for Geriatric Disorders, Xiangya Hospital, Central South University, Changsha, Hunan, China; ^3^Department of Radiology, Xiangya Hospital, Central South University, Changsha, Hunan, China

**Keywords:** major depressive disorder, radiology, bibliometric analysis, Latent Dirichlet allocation, machine learning

## Abstract

**Objective:**

This study aims to construct and use natural language processing and other methods to analyze major depressive disorder (MDD) and radiology studies’ publications in the PubMed database to understand the historical growth, current state, and potential expansion trend.

**Methods:**

All MDD radiology studies publications from January 2002 to January 2022 were downloaded from PubMed using R, a statistical computing language. R and the interpretive general-purpose programming language Python were used to extract publication dates, geographic information, and abstracts from each publication’s metadata for bibliometric analysis. The generative statistical algorithm “Latent Dirichlet allocation” (LDA) was applied to identify specific research focus and trends. The unsupervised Leuven algorithm was used to build a network to identify relationships between research focus.

**Results:**

A total of 5,566 publications on MDD and radiology research were identified, and there is a rapid upward trend. The top-cited publications were 11,042, and the highly-cited publications focused on improving diagnostic performance and establishing imaging standards. Publications came from 76 countries, with the most from research institutions in the United States and China. Hospitals and radiology departments take the lead in research and have an advantage. The extensive field of study contains 12,058 Medical Subject Heading (MeSH) terms. Based on the LDA algorithm, three areas were identified that have become the focus of research in recent years, “Symptoms and treatment,” “Brain structure and imaging,” and “Comorbidities research.”

**Conclusion:**

Latent Dirichlet allocation analysis methods can be well used to analyze many texts and discover recent research trends and focus. In the past 20 years, the research on MDD and radiology has focused on exploring MDD mechanisms, establishing standards, and constructing imaging methods. Recent research focuses are “Symptoms and sleep,” “Brain structure study,” and “functional connectivity.” New progress may be made in studies on MDD complications and the combination of brain structure and metabolism.

## Introduction

Major depressive disorder (MDD) is an affective psychosis characterized by depression with symptoms such as sleep disturbance, anxiety, and physical discomfort (1, 2). MDD is one of the leading causes of disability and suicide, with more than 800,000 suicides worldwide (3). It is the fourth leading disabling disease globally (4). To make matters worse, the pathogenesis of MDD is not well understood, and treatment options are limited (5). Furthermore, under the influence of the COVID-19 pandemic, the number of MDD patients and the degree of deterioration will increase (6). MDD will cause great trouble to human health in the foreseeable future.

Current research shows that MDD is closely related to brain structure and function changes. With the development of radiology, significant progress has been made in exploring the neurobiological mechanisms of MDD (7). Radiology can not only use a variety of non-invasive methods to detect and analyze brain structure but also explore MDD brain function and brain connectivity through functional magnetic resonance imaging (fMRI) (8). In addition, techniques such as positron emission tomography (PET) and magnetic resonance spectroscopy (MRS) can track a variety of substances, thereby revealing the relationship between depression and brain metabolism (9, 10). Therefore, radiology has an essential function as a diagnostic and research tool to explore the pathogenesis of MDD.

The text volume of MDD and Radiology publications worldwide has exceeded tens of millions of words, and it is impossible to extract the information from the papers by reading. The correlation between papers, continuity, and other complex internal correlation information has exceeded the scope of human subjective understanding. Natural language processing (NLP) is a machine learning technology tool specializing in text processing (11). It has been widely used in medical research. It can analyze many texts, extract research topics, and visually facilitate researchers to understand massive texts and extract internal correlations (12, 13).

This study aimed to perform a bibliometric analysis of all publications related to depression and radiology over the past 20 years. Further build and use NLP methods and various algorithms, such as Latent Dirichlet allocation (LDA), to discover changes in research focus and future predictions. This research aims to use the NLP method and multiple algorithms, such as LDA, to conduct a bibliometric analysis of all publications related to depression and radiology in the past 20 years. This study hopes to extract relevant research focus, discover changes in research content, predict possible future breakthroughs direction, and summarize the research of the last 20 years.

## Materials and methods

### Research design

The study design was based on compliance with the fundamental principles of bibliometrics (14, 15). The study employed a two-stage structured approach to bibliometric analysis and visual assessment of published scientific literature. The PubMed database is a biomedical specialty database containing compound search strategies and is a free, publicly available database. The PubMed database is used for research purposes, and PubMed contains Application Programming Interface (API) that can export abstracts. In addition, all MDD and its radiology-related publications must be searched as thoroughly as possible for research purposes.

### Inclusive and exclusive criteria

**FIGURE 1:**
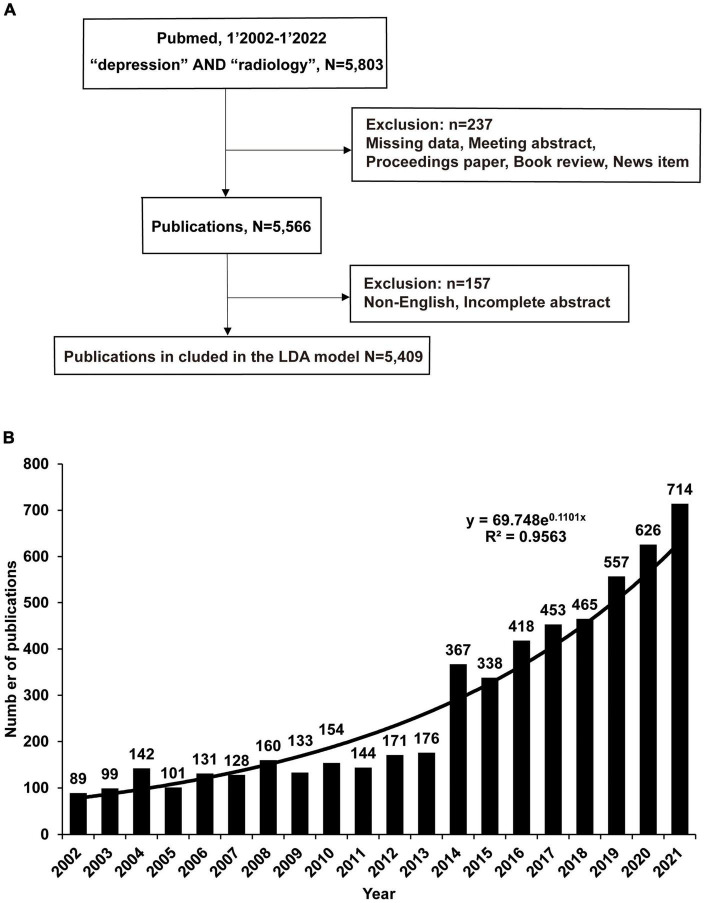
The number of publications on radiology and depression has increased rapidly over the past 20 years. **(A)** Using the search terms depression and radiology in the PubMed database, download publications through the R pubquery package. Missing data or when the publication was a meeting abstract, proceedings paper, a correction, a book review, or a news item were manually excluded, and finally, 5,566 publications were included in the general analysis. LDA analyzed 5,409 publications. **(B)** Publications analyzed by LDA, Python. Data were visualized using Excel. The number of publications is shown each year, and y = 69.748e^0.1101x^ is the fitted function.

Table 1 shows the steps to obtain the complete raw data in the PubMed database. All MDD Radiology-related publications were published between January 1, 2002, and January 1, 2022, resulting in 5,803 publications. Missing data, conference abstracts, conference proceedings, book reviews, and news items were excluded, and ultimately 5,566 publications were included in the bibliometric analysis (Figure 1). Details of inclusion and exclusion are shown in Table 2. After excluding publications with the most non-English and incomplete abstracts, the final 5,406 publications were analyzed by the LDA algorithm to obtain changes in the focus of research topics in publications in this field and their correlations.

**TABLE 1:** Major depressive disorder (MDD) and radiology publications assortment steps.

Exploration steps	Query on pubMed	Description
1	Depression	”depressed”[All Fields] OR “depression”[MeSH Terms] OR “depression”[All Fields] OR “depressions”[All Fields] OR “depression’s”[All Fields] OR “depressive disorder”[MeSH Terms] OR (”depressive”[All Fields] AND “disorder”[All Fields]) OR “depressive disorder”[All Fields] OR “depressivity”[All Fields] OR “depressive”[All Fields] OR “depressively”[All Fields] OR “depressiveness”[All Fields] OR “depressives”[All Fields]
2	AND	Stop word: and
3	Radiology	”radiology”[MeSH Terms] OR “radiology”[All Fields] OR “radiography”[MeSH Terms] OR “radiography”[All Fields] OR “radiology’s”[All Fields]
4	Data limiation	(2002:2022[pdat])

**TABLE 2:** Inclusive and exclusive criteria.

Parameter of selection of a publication	Inclusion criterion	Exclusion criterion	Rationale for inclusion–exclusion
Language	English	All other languages	The working language of LDA algorithm is English.
Year	2002–2021	Publications before 2021 and after 2002	2022’s publications cannot be included because it has not been fully published
Publication Type	All	Missing data, meeting abstract, proceeding paper, book review, news item	In addition to incomplete content, try to include research articles and review
Funding sponsor	All	No exclusion	This parameter does not affect the selection criterion
Affiliation	All	No exclusion	This parameter does not affect the selection criterion
Funding	All	No exclusion	This parameter does not affect the selection criterion
Country	All	No exclusion	Publication from each country has its significance

### Retrieval and visualization tools

The R language^1^ (version: 4.1.3) was used to access the PubMed database through the pubquery package and download publications limited to publication dates from January 1, 2002, to January 1, 2022. In order to include the total number of possible publications, the most comprehensive search strategies “depression” and “radiology” were adopted and the database was eventually downloaded. For specific search terms, please refer to the Supplementary material. Tables and visualizations are based on R and Excel (Microsoft Corporation, Redmond, WA, USA). Search and download code is available in R through the easyPubMed package.^2^

### Latent Dirichlet allocation and algorithms and analytical methods

After the publication acquisition, the publication was processed and analyzed based on the flowchart in Figure 1A. Guido van Rossum created Python in the Netherlands in the early 1990s at Stichting Mathematisch Centrum. Python provides efficient data structures and simple and effective object-oriented programming and is widely used in machine learning and NLP. Python^3^ (version 3.10.4) extracted publication data, including publication year, geographic information, abstract, study type, and Medical Subject Heading (MeSH) terms. MeSH terms are a controlled vocabulary dictionary produced by the National Library of Medicine.^4^ The PubMed database uses MeSH terms to summarize the topic content of each publication, and MeSH terms were used to conduct a preliminary analysis of the development of research topics. LDA is an unsupervised topic model algorithm built according to previous research methods (16). LDA identifies abstracts for all publications and sets the number of identified topics to 50. Criteria for topic number selection are based on appropriate perplexity, redundancy, and legibility levels. LDA is used to identify more specific research topics based on algorithmically computed topic probabilities and the opinions of all authors, ultimately manually determining the topic of each article. Heatmaps were generated in R to describe research topics and publication dates. Cluster analysis was performed using the Louvain algorithm in Gephi software^5^ (version 0.9.4) to build topic networks and determine relationships between topics. The two topics with the highest attribution probability in each article were used to calculate the number of co-occurrences in each document (17). The LDA-related code is available in the Supplementary material (see footnote 2).

## Results

### The number of publications of radiology and major depressive disorder-related research is increasing every year

From 2002 to 2022, 5,566 relevant publications were included, as shown in the flowchart (Figure 1A). A total of 237 publications were manually excluded, and the exclusion criteria were missing data if the publications were conference abstracts, conference papers, corrections, book reviews, or news items. The number of remaining 5,566 publications was further reduced due to the manual exclusion of non-English publications or publications with incomplete abstracts, resulting in a remaining 5,409 publications. The LDA bibliometric modeling approach further analyzed these selected publications.

Eighty-nine publications related to depression and radiology were found in 2002, which increased to 714 publications in 2021 (Figure 1B). An average of 278 publications were published annually, representing an average annual growth rate of 14.4%. Using exponential function fitting found that *R*^2^ = 0.96, and the composite function y = 69.748e^0^.^1101x^ can describe the number of publications well. It is expected that there will be 750 and 840 publications in 2022 and 2023.

**FIGURE 2:**
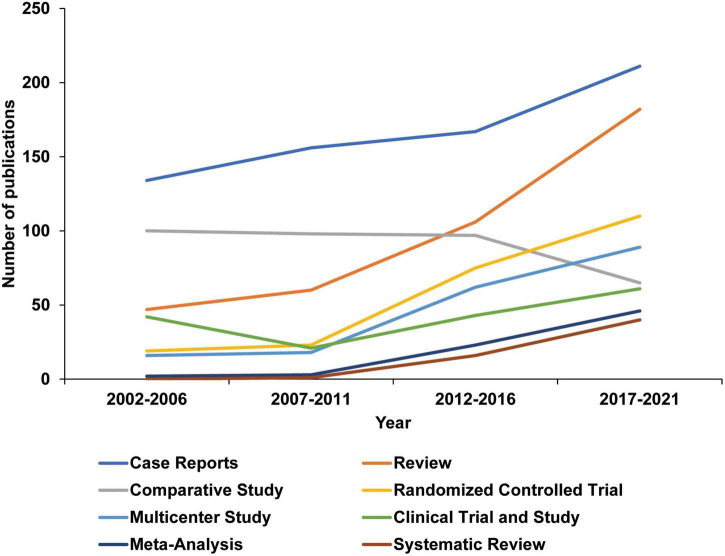
Changes in depression and radiology publication types over the last 20 years. Publication types are defined by the PubMed database and extracted using Python.

In order to further explore the research types, eight categories based on the classification of publications provided by the PubMed database were identified: Case Reports, Review, Comparative Study, Randomized Controlled Trial, Multicenter Study, Clinical Trial and Study, Meta-Analysis, Systematic Review. In the past 20 years, the number of all types of research has increased significantly, the most notable is Review, from 47 in 2002–2006 to 182 in 2017–2021(Supplementary Table 1 and Figure 2). On the other hand, the number of Randomized Controlled Trial published from 19 in 2002–2006 to 110 in 2017–2021, and the Multicenter Study publications also increased considerably, indicating that a large number of clinical trials in this field have been published. Based on the data, it can be assumed that more than 130 clinical trials are in progress.

### High-cited reviews focus on reviews related to pathogenic mechanisms

Review plays a role in summarizing a branch research direction in MDD and radiology research. High citations often mean a more significant impact and a link to research. In addition, the number of internal citations by other selected publications is considered to be a critical performance in the field. A total of 395 publications were extracted and identified as reviews. The essential characteristics of the number of publications are shown in Figure 1. The average number of publications is 19.75 per year, and it can be found that the number of publications has increased significantly since 2016, and it is expected that the number of papers published in 2022 maybe 80. Table 3 shows the 10 most internally cited reviews and their total citation counts. Among them, Bolay H’s migraine model research is the most cited internally, with 10 internal citations (18), and Julian’s inconsistency in meta-analyses research, with total citations of 36,995.

**TABLE 3:** Top 10 review publications of major depressive disorder (MDD) and radiology based on internal citations.

Reference title	DOI	Title	Internal citation	Total citation
Nat Med. 2002 Feb;8(2):136-42	10.1038/nm0202-13610.1038/nm0202-136	Intrinsic brain activity triggers trigeminal meningeal afferents in a migraine model (18)	10	825
BMJ. 2003 Sep 6;327(7414):557-60	10.1136/bmj.327.7414.55710.1136/bmj.327.7414.557	Measuring inconsistency in meta-analyses (19)	9	36,995
J Cereb Blood Flow Metab. 2009 Sep;29(9):1517-27	10.1038/jcbfm.2009.7310.1038/jcbfm.2009.73	Persistent increase in oxygen consumption and impaired neurovascular coupling after spreading depression in rat neocortex (24)	9	151
Nat Med. 2011 Apr;17(4):439-47	10.1038/nm.233310.1038/nm.2333	The role of spreading depression, spreading depolarization and spreading ischemia in neurological disease (26)	9	704
PLoS Med. 2009 Jul 21;6(7):e1000097	10.1371/journal.pmed.100009710.1371/journal.pmed.1000097	Preferred reporting items for systematic reviews and meta-analyses: the PRISMA statement (23)	9	24,250
Ann Neurol. 2010 Feb;67(2):221-9	10.1002/ana.2187110.1002/ana.21871	Microemboli may link spreading depression, migraine aura, and patent foramen ovale (25)	8	200
J Cereb Blood Flow Metab. 2011 Jan;31(1):17-35	10.1038/jcbfm.2010.19110.1038/jcbfm.2010.191	Clinical relevance of cortical spreading depression in neurological disorders: migraine, malignant stroke, subarachnoid and intracranial hemorrhage, and traumatic brain injury (27)	8	389
J Clin Invest. 2009 Jan;119(1):99-109	10.1172/JCI3605910.1172/JCI36059	Genetic and hormonal factors modulate spreading depression and transient hemiparesis in mouse models of familial hemiplegic migraine type 1(21)	8	195
Biol Psychiatry. 2009 May 1;65(9):732-41	10.1016/j.biopsych.2008.11.02910.1016/j.biopsych.2008.11.029	Inflammation and its discontents: the role of cytokines in the pathophysiology of major depression (22)	7	2,335
Brain. 2006 Mar;129(Pt 3):778-90	10.1093/brain/awh71610.1093/brain/awh716	Cortical spreading depression and peri-infarct depolarization in acutely injured human cerebral cortex (20)	7	299

High citation review mainly covers the mechanism of acute injury, inflammation, genetic factors, trauma, vascular microembolism, ischemia, and migraine model on MDD, and also includes the criteria and discussion of systematic review methods (18–27). It shows that high-cited reviews focus on reviews related to pathogenic mechanisms, and this trend will continue (Figure 2 and Supplementary Figure 1). At the same time, combined with Meta-analysis and the increase in the number of publications of clinical trials, more systematic review studies based on trials and data may appear in the future to guide research on MDD and radiology-related mechanisms.

### High-cited publications focus on establishing criteria for the diagnosis of major depressive disorder and constructing imaging methods

High-cited publications are considered critical publications for research in related fields. Internal citations were also given more importance among the publications included in the analysis. It was found that the total number of citations exceeded 60,000, of which 105 were internally cited the most, accounting for 2% of the total included publications (Table 4 and Supplementary Table 2). The most internally cited is N Tzourio-Mazoyer’s macroscopic brain anatomy by MRI, totaling 11,042 citations (28). The results suggest that the most cited publication can be roughly divided into two categories. The first research focuses on the establishment of diagnostic criteria for depression. Multiple research has established diagnostic criteria, including scales and grading methods used by physicians, and the diagnosis of MDD has been further strengthened based on these definitive studies (29–33).

**TABLE 4:** Top 10 cited publications of major depressive disorder (MDD) and radiology.

Reference title	DOI	Title	Internal citation	Total citations
Neuroimage. 2002 Jan;15(1):273-89	10.1006/nimg.2001.097810.1006/nimg.2001.0978	Automated anatomical labeling of activations in SPM using a macroscopic anatomical parcellation of the MNI MRI single-subject brain (28)	109	11,042
Biol Psychiatry. 2007 Sep 1;62(5):429-37	10.1016/j.biopsych.2006.09.02010.1016/j.biopsych.2006.09.020	Resting-state functional connectivity in major depression: abnormally increased contributions from subgenual cingulate cortex and thalamus (50)	84	1,506
JAMA Psychiatry. 2015 Jun;72(6):603-11	10.1001/jamapsychiatry.2015.007110.1001/jamapsychiatry.2015.0071	Large-Scale Network Dysfunction in Major Depressive Disorder: A Meta-analysis of Resting-State Functional Connectivity (36)	76	941
Neuroimage. 2006 Jul 1;31(3):968-80	10.1016/j.neuroimage.2006.01.02110.1016/j.neuroimage.2006.01.021	An automated labeling system for subdividing the human cerebral cortex on MRI scans into gyral based regions of interest (35)	68	6,651
Neuron. 2002 Jan 31;33(3):341-55	10.1016/s0896-6273(02)00569-X10.1016/s0896-6273(02)00569-X	Whole brain segmentation: automated labeling of neuroanatomical structures in the human brain (34)	68	5,508
Ann N Y Acad Sci. 2008 Mar;1124:1-38	10.1196/annals.1440.01110.1196/annals.1440.011	The brain’s default network: anatomy, function, and relevance to disease (31)	63	6,922
Neuroimage. 2007 Oct 15;38(1):95-113	10.1016/j.neuroimage.2007.07.00710.1016/j.neuroimage.2007.07.007	A fast diffeomorphic image registration algorithm (30)	62	5,255
Neuroimage. 2012 Feb 1;59(3):2142-54	10.1016/j.neuroimage.2011.10.01810.1016/j.neuroimage.2011.10.018	Spurious but systematic correlations in functional connectivity MRI networks arise from subject motion (33)	61	4,631
Neuroimage. 2004;23 Suppl 1:S208-19	10.1016/j.neuroimage.2004.07.05110.1016/j.neuroimage.2004.07.051	Advances in functional and structural MR image analysis and implementation as FSL (29)	55	8,671
Proc Natl Acad Sci U S A. 2010 Jun 15;107(24):11020-5	10.1073/pnas.100044610710.1073/pnas.1000446107	Resting-state functional MRI in depression unmasks increased connectivity between networks via the dorsal nexus (32)	55	768

The other is the detection of MDD brain regions in radiology methods, such as the cerebral cortex, Large-Scale Network Dysfunction, and the use of influence studies to study MDD and to determine its correlation and build a scale (34–36). These highly cited papers have played a vital role in clinical research, diagnosis, and image registration algorithm. These two types of publications will be cited more in the future based on the research results, these studies have found a precise and reproducible research pattern in the complex relationship between MDD and imaging to a certain extent, and the number of citations will be increased in the foreseeable future.

### Hospitals play an essential role in major depressive disorder and radiology research

The organization of a publication can often indicate the publication’s research location and characteristics. Because multiple research institutions often accompany publications. The PubMed database provides the detailed organization of each author of the manuscript. The publications’ first organization was analyzed to facilitate statistics and refine the author’s specific department. The organization that published the most papers was Huaxi MR Research Center from West China Hospital, Sichuan University, China, with 25 publications, followed by the Department of Psychiatry, University of Munster, Munster, Germany, with 12 publications (Table 5). It was found that most research organizations were hospitals, especially radiology departments, accounting for more than 70%. This suggests that a hospital is an essential research organization to study MDD and radiology departments because they are more likely to obtain support from clinical departments and patients, and colleagues in the radiology department can assist in scientific research, diagnosis, and follow-up. It also suggests that further scientific research departments, fundamental neuroscience research departments, may be able to conduct more profound research through better cooperation with hospitals.

**TABLE 5:** Top 10 organizations with the most publications.

Organizations and departments	Publication number	Rank
Huaxi MR Research Center (HMRRC), Department of Radiology, West China Hospital of Sichuan University, Chengdu, China	25	1
Department of Psychiatry, University of Munster, Munster, Germany	12	2
Pain Relief and Palliative Care Unit, Department of Radiology, Areteion Hospital, School of Medicine, University of Athens, Athens, Greece	10	3
Department of Psychological & Brain Sciences, Washington University in St. Louis, USA	9	4
Department of Psychiatry, Washington University in St. Louis, USA	7	5
Department of Radiology, The Second Xiangya Hospital, Central South University, Changsha, China	7	6
Department of Psychiatry, Hotchkiss Brain Institute, University of Calgary, Calgary, Alberta, Canada	6	7
Department of Radiology, The First Affiliated Hospital of Anhui Medical University, Hefei, China	6	8
Department of Psychiatry, University of Occupational and Environmental Health, Kitakyushu, Japan	5	9
Neurovascular Research Laboratory, Department of Radiology, Massachusetts General Hospital, Harvard Medical School, Charlestown, MA, USA	5	10

### The United States, China, and Japan have the highest number of publications in the field of depression and radiology

**FIGURE 3:**
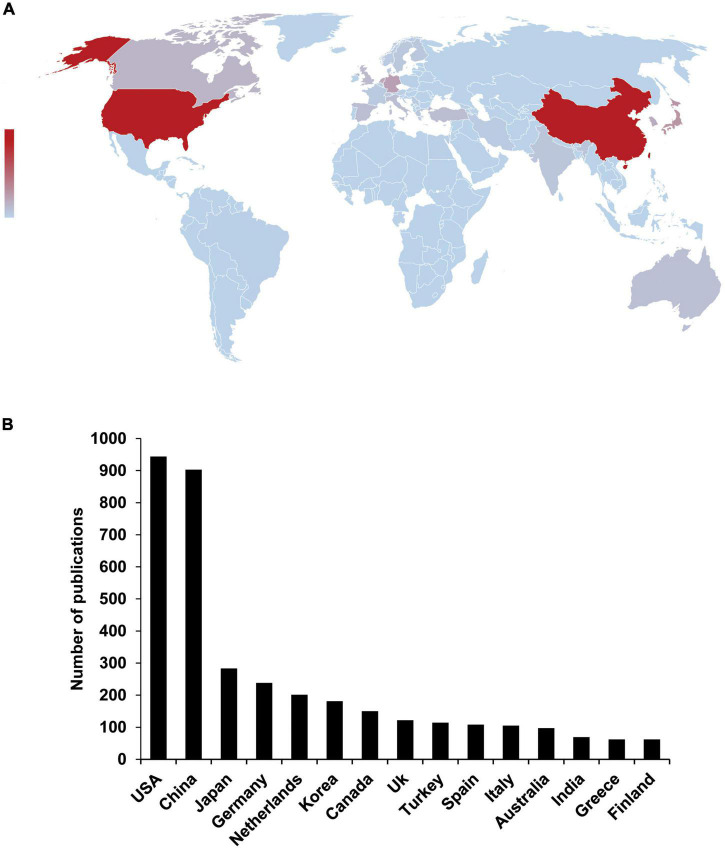
Regional differences in the research status of global depression and radiology. **(A)** The map shows the global distribution of depression and radiology publications over 20 years. Country information was extracted based on publication affiliation. Countries or regions were drowned with publications; the darker the color, the greater the number of publications. The number of publications in the Northern Hemisphere is much higher than in the Southern Hemisphere. **(B)** Top 15 countries with the most published depression and radiology studies.

Next, the author’s affiliation information was analyzed to study research institutions’ geographic distribution. Institutions in 76 countries or regions worldwide were identified, most located in the northern hemisphere, participated in relevant research and published manuscripts (Figure 3A). Among all publications, the USA, China, Japan, Germany, and the Netherlands accounted for 21.5, 20.6, 6.4, 5.4, and 4.6%, respectively. The top 10 countries with the most significant publications accounted for 73.9% of all publications (Figure 3B). The study found that Southeast Asia and Africa contributed less than 5% of the total publications. Based on geographic data, the vast majority of the world’s population participates in relevant research, but there are considerable differences in published data.

### Medical Subject Heading terminology research suggests that “diagnostic imaging,” “MRI,” and “physiopathology” are the most studied areas

**FIGURE 4:**
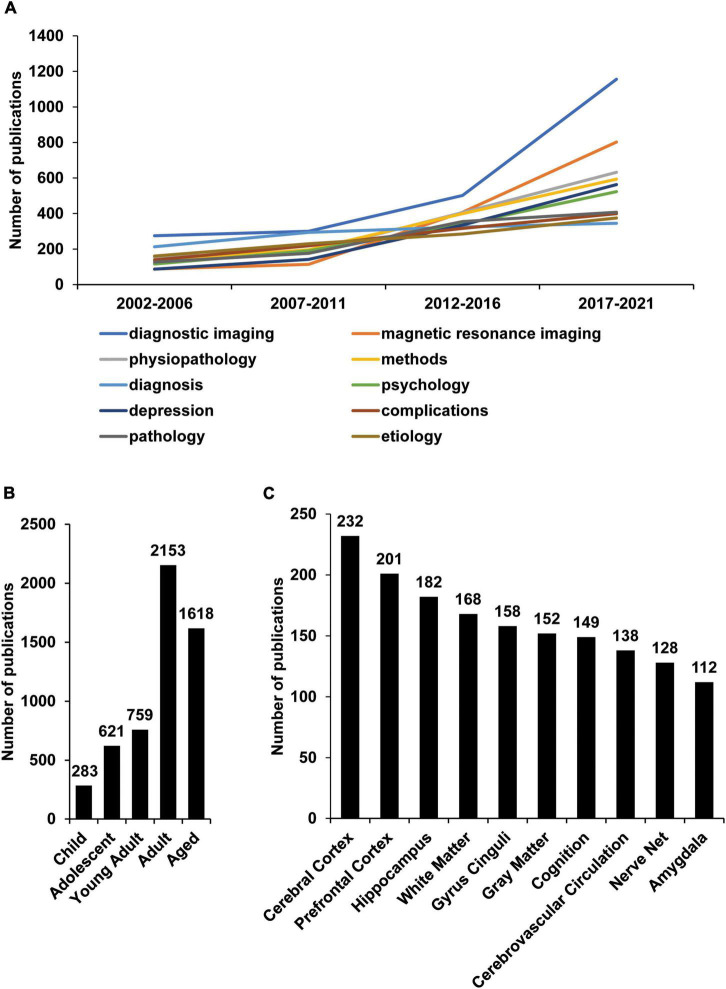
Research changes in depression and radiology through a study of MeSH terminology. R extracted significant MeSH terms from publications listed in the PubMed database according to the publication topic. Each publication is defined by the PubMed database, which can indicate the general content of the paper research. Each publication contains multiple MeSH terms, which extracted by R and Python. **(A)** Ten of the most widely studied MeSH terms and their number (No.) of publications per year. “diagnostic imaging,” “MRI,” and “physiopathology” were the most frequently studied research fields in the last 20 years. **(B)** The number of publications studying different age groups. Adults is the most studied group. **(C)** The most studied brain structures and their number of publications. The Cerebral Cortex, Prefrontal Cortex, and Hippocampus are the most studied brain structures.

To further explore the changes in research directions, the MeSH terms of all publications were extracted and analyzed, which can partially reflect research topics. 5,561 MeSH were studied, and all MeSH terms have been studied 85,181 times, indicating that the research scope of depression and radiology is extensive. Diagnostic imaging, magnetic resonance imaging, and physiopathology have been studied the most, reaching 1156, 802, and 632 times (Figure 4A). Interestingly, 2,153 and 1,618 studies on the Adult and Aged populations in age-related MeSH terms were found, indicating that researchers conducted the most studies on adults (Figure 4B). Regarding brain structure, 10 MeSH terms with the most research on brain structure were extracted, among which Cerebral Cortex, Prefrontal Cortex, and Hippocampus are the most studied. Furthermore, the difference in the number of studies on other structures is not apparent, but in terms of the number of studies, these structures may be the most closely related to depression and radiology and suggest that they are the most concerned by researchers (Figure 4C).

### Latent Dirichlet allocation identified the main areas of depression and radiology are“Symptoms and Treatment,” “Brain structure and Imaging,” and “Comorbidities research”

**FIGURE 5:**
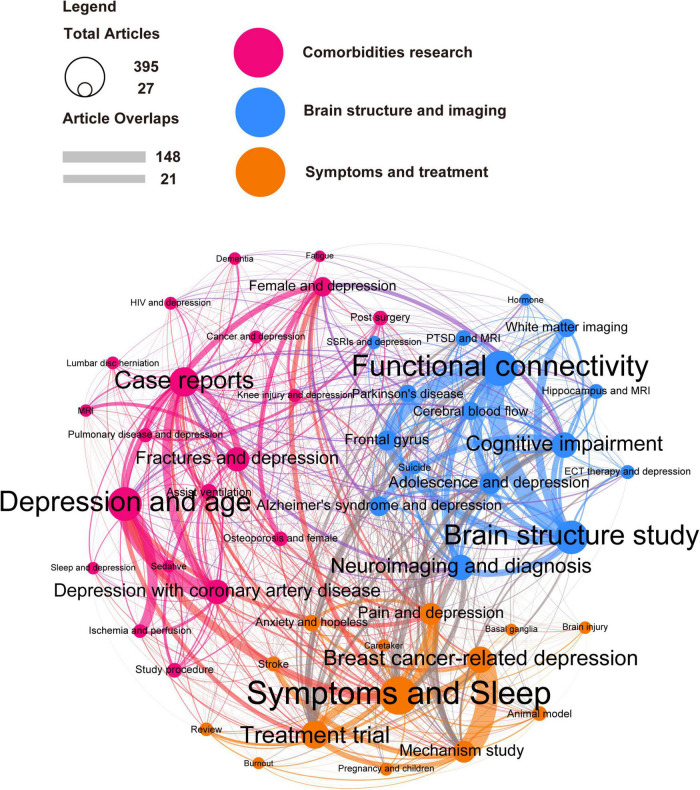
Latent Dirichlet allocation (LDA) identified the main areas of depression and radiology are “Symptoms and Treatment,” “Brain Structure and Imaging,” and “Comorbidities research.” The LDA algorithm was used to analyze publications from the last 20 years. from which 50 topics were identified and classified into three broad categories: “Symptoms and Treatment” (orange), “Brain Structure and Imaging” (blue), and “Comorbidities research” (rose-red). The circle size represents the number of publications contained in the research topic. For example, the algorithm found that the topic “Depression and age” contains 395 publications. The line thickness between the circles represents the degree of overlap between the two research topics, such as the 148 publications with the highest overlap in “Breast cancer-related depression” and “Mechanism study.”

To further identify a specific research focus, the topic model LDA algorithm were constructed to generate relevant topics based on the content of each publication. To express the relationships between interrelated topic clusters and underlying topics, topic network analysis and visualization analysis using LDA and Leuven algorithms were built. The algorithm divides the past 20 years of research into these three research clusters, “Symptoms and Treatment,” “Brain Structure and Imaging,” and “concurrent disease research” (Figure 5). The line thickness between the circles indicates the correlation between the two topics. For example, the orange marker cluster “Symptoms and Treatment” can be subdivided into “Symptoms and Sleep,” “Treatment trial,” “Treatment trial,” “Review,” and other areas, with the circle size determined by the number of publications. Symptoms and Sleep are the topics of most significant interest in research. As the connecting line indicates, there is a strong relationship between “Breast cancer-related depression” and “Mechanism study,” indicating that the links are closely related. The blue cluster “Brain Structure and Imaging” covers branches including “Brain structure study” and “Functional connectivity,” “Neuroimaging and diagnosis,” “Cognitive impairment,” “Adolescence and depression,” “SSRIs and depression.” This cluster can be interpreted as the study of the depressive brain by multiple imaging techniques. The rose-red cluster “Comorbidities research” includes its branches “Depression and age,” “Case reports,” “Depression with coronary artery disease,” “Fractures and depression,” “HIV and depression,” and other research. This cluster suggests that coronary heart disease, fractures, and lung diseases are closely related to depression and radiology. In conclusion, research on depression and radiology covers a wide range of clinical, brain structures, and comorbidities of depression.

### Latent Dirichlet allocation results suggest that Symptoms and Sleep, Brain structure study, and functional connectivity are recent research focus

**FIGURE 6:**
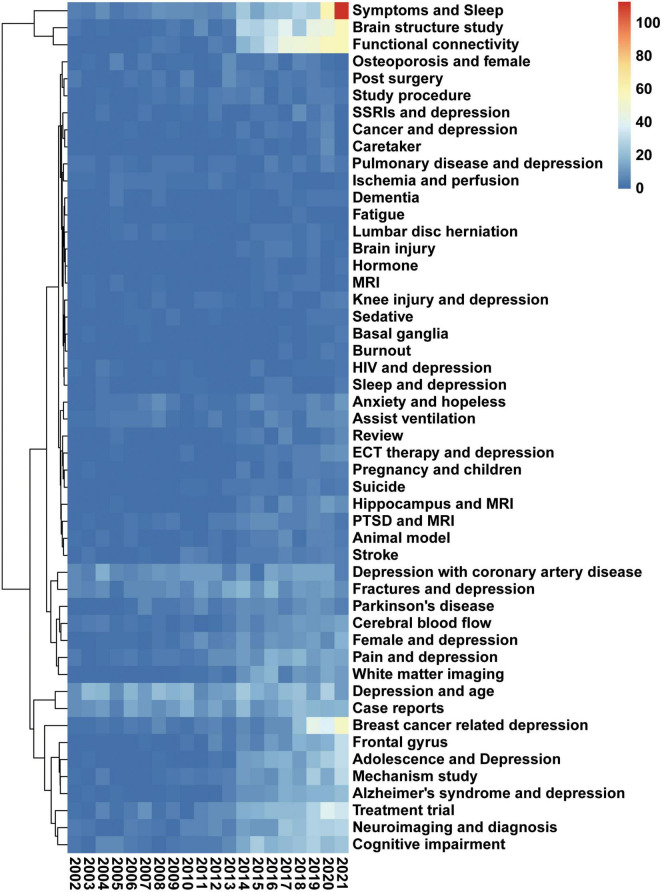
“Symptoms and sleep,” “Brain structure study,” and “functional connectivity.” The heatmap presents the change of 50 research topics over the past 20 years. Data were generated by the use of the LDA algorithm. The abscissa represents the year, the ordinate represents the topics, and the color brightness represents the number of publications and reflects the shift in research focus. The legend represents the number of publications per color.

Latent Dirichlet allocation results were visualized with a heatmap to understand further the evolution of the general research focus in depression and radiology. The heatmap shows 50 research topics and annual changes in publications from 2002 to 2021 (Figure 6). Publication numbers in the three areas of Symptoms and Sleep, Brain structure study, and functional connectivity have increased dramatically. Therefore, based on the number of publications, these three topics may be the focus of current and future research.

## Discussion

Machine learning and NLP were used to analyze 5,566 PubMed publications in depression radiology research from 2002 to 2022. The results from multiple perspectives were analyzed and visualized, including publication numbers, geographic information, MeSH terms, and topic model algorithms. This is the first such analysis in this field. Over the past 20 years, the number of depression and radiology research publications has increased from 3,359 in 2002 to 10,145 in 2021. The top-cited publications were 11,042, and the highly-cited publications focused on improving diagnostic performance and establishing imaging standards. Hospitals and radiology departments take the lead in research and have an advantage. The extensive field of study contains 12,058 MeSH terms. Many regions worldwide participated in the study, especially the Northern Hemisphere. In the past 20 years, depression radiology research has mainly focused on “Symptoms and Treatment,” “Brain Structure and Imaging,” and “Comorbidities research.” Significantly the research focus has changed in recent years, and the current research focus is expected to be on symptoms and sleep, brain structure study, and functional connectivity.

Machine learning and NLP often analyze large amounts of data resulting in difficulty understanding the results, also known as inexplicability. The LDA algorithm was used, an unsupervised algorithm that does not require labels and training sets for the analyzed text and can be used on personal computers. At the same time, multiple visualization software and the opinions of multiple professional authors were combined, striving to be able to explain the machine learning results. In addition, bibliometric analysis can be performed with a few software, such as Bibliographic Items Co-Occurrence Matrix Builder (BICOMB), VOSviewer, and CiteSpace (37, 38). However, these software cannot analyze a large amount of text processing, so a new analysis method was built.

This study found that the radiology study of depression includes many studies on the comorbidities of depression, such as breast cancer, fracture, coronary artery disease, Alzheimer’s disease (AD), and respiratory system diseases, accounting for about 20% of the total publications. About 668 publications are Case Reports. On the one hand, many patients cannot accurately identify their depression. About 50–70% of patients are found to suffer from depression because of comorbidities (39). Many studies have pointed out that depression, coronary artery disease, and diabetes have mutually reinforcing risks (40, 41). On the other hand, these patients often undergo various radiological examinations to identify lesions and assess disease status, which has increased people’s understanding of depression from another perspective. Depression can be observed in 50% of AD patients, and MRI revealed widespread brain atrophy and disruption of white matter integrity and cognitive impairment, and higher depression severity (42). this study found breast cancer; a significant correlation with studies on mechanisms of depression, with a weight of 148. In the further analysis, results shows found that there is a strong correlation between “women,” “age,” “lack of sleep,” and “COVID-19.” Breast cancer radiology studies uncover most underlying mechanisms underlying a functional dorsal attention network (DAN). Changes in breast cancer patients without chemotherapy may be due to insufficient frontoparietal neural activity to drive DAN and may be related to the effects of neuropsychological distress. Suggesting that older women and breast cancer patients are more vulnerable to depression (43). In conclusion, depression and comorbidities can promote the study of depression by imaging from another perspective.

The brain structure study lists the top 10 most studied brain structures in depression radiology in the last 20 years. The top three are the cerebral cortex, prefrontal cortex, and hippocampus, with 232, 201, and 182 publications. This may mean that researchers have the most understanding of the brain structure of depression from a radiology perspective. Based on providing data from 10 brain lesions, building a scoring system based on big data in radiology may be a new way to assess depression (44). In addition, LDA analysis found that under the research topic of functional connectivity, the number of publications exceeded 300, and the number of publications in recent years has shown a clear upward trend. This shows that researchers’ understanding of depression from a radiological perspective begins to deepen the connection between brain regions. A recent study using machine learning found that the co-activation pattern (CAP) of the salience network (SN) in MDD patients has a transitional enhancement to CAP (45). The results showed that the flexibility of the network was enhanced in patients. The study also found reduced spatial consistency and persistence of the default mode network (DMN) in patients, suggesting reduced variability and stability in patients with MDD (46). Based on this, it is speculate that this direction will be the focus of research in the future, and it is very likely to combine brain metabolomics and brain function to discover new critical therapeutic targets.

Radiology is now widely used to diagnose, treat, evaluate, and research MDD. More than 400 clinical trials of various types have been published, covering a variety of diseases, such as tumors, and radiological evaluation of the treatment of post-traumatic depression. There are currently more than 400 publications related to clinical trials, covering a variety of diseases, such as cancer, and radiological evaluation of the treatment of post-traumatic depression. However, many keywords related to primary medical institutions and related research topics, Caregiver and Primary care, were found in the diagnosis of MDD. Harman’s study shows that adults with clinically significant unipolar depression tend to present to primary care rather than psychiatry, and 50% were not screened or evaluated for depression (47, 48). Recent studies have identified a potential link between connectome gradient disruption and the sensory-cognitive impairment accompanying MDD in MDD patients from the functional connectome and DMN. With the aid of various new radiological tools such as fMRI and MRS for diagnosis, better radiological diagnostic and predictive criteria will be constructed for the medication response of MDD patients.

In addition, sleep disorders and sleep-wake disorders as MeSH terms appeared in more than 500 publications. Studies have found that more than 70% of MDD patients suffer from sleep disturbances. Radiology plays a vital role in sleep and MDD-related research—especially the application of recent radiology technology. The current study confirmed that amygdala-based rtfMRI-NF training altered intrinsic functional centers remodeled abnormal functional connectivity caused by insomnia, and improved sleep in CID patients. These findings contribute to understanding of the neurobiological mechanisms of rtfMRI-NF in the treatment of insomnia (8). There has been a rapid increase in the number of papers published in this field in recent years, and there may be new advances in research predicting new technologies for sleep disorder research.

As for the limitations, firstly, all the included publications are from the PubMed database, and the publications only include English papers. The data included in PubMed is relatively complete; it may still be biased (49). Secondly, road search terms were used to include the most comprehensive publications possible, which may lead to bias. This study uses an analytical approach from an NLP perspective and superficial insights into depression and radiology research topics. However, machine learning and NLP are new tools for scientists to extract objective and comprehensive clues from large amounts of data.

## Conclusion

Latent Dirichlet allocation analysis methods can be well used to analyze many texts and discover recent research trends and focus. In the past 20 years, the research on MDD and radiology has focused on exploring MDD mechanisms, establishing standards, and constructing imaging methods. Recent research focuses are “Symptoms and sleep,” “Brain structure study,” and “functional connectivity.” New progress may be made in studies on MDD complications and the combination of brain structure and metabolism.

## Data availability statement

The original contributions presented in this study are included in the article/Supplementary material, further inquiries can be directed to the corresponding author.

## Author contributions

KW collected and analyzed the data and wrote the manuscript. FT, ZZ, and LK provided extensive guidance and feedback on the manuscript. All authors contributed to the design of the study and have read and approved the final manuscript.

## Footnotes

1
https://www.r-project.org/

2
https://cran.r-project.org/web/packages/easyPubMed/index.html

3
https://www.python.org/

4
https://www.ncbi.nlm.nih.gov/mesh

5
https://gephi.org/
