# Supplementary material for: Exploring changes in depression and radiology-related publications research focus: A bibliometrics and content analysis based on natural language processing
Source: Front Psychiatry. 2022 Nov 30;13:978763. doi: 10.3389/fpsyt.2022.978763 (PMC9748702; doi:10.3389/fpsyt.2022.978763)
Supplement: Supplementary file 1 [file Data_Sheet_1.docx]

**Supplement**

**Exploring Changes in Depression and Radiology-related Publications Research Focus: A Bibliometrics and Content Analysis Based on Natural Language Processing**

Kangtao Wang, Fengbo Tan, Zhiming Zhu, Lingyu Kong

**Table of Content**

**Supplemental information 1** Search terms

**Table S1.** Changes in the number of publication types from 2002 to 2022.

**Table S2.** Top 10 most citation for included publications.

**Figure S1.** Number of publications of Review in MDD and radiology studies

**Supplemental information 2** Code of Latent Dirichlet allocation methods

**Supplemental information 1** Search terms

"depressed" [All Fields] OR "depression" [MeSH Terms] OR "depression" [All Fields] OR "depressions" [All Fields] OR "depression s" [All Fields] OR "depressive disorder"[MeSH Terms] OR ("depressive"[All Fields] AND"disorder"[All Fields]) OR"depressive disorder" [All Fields] OR"depressivity" [All Fields] OR"depressive" [All Fields] OR "depressively" [All Fields] OR "depressiveness"[All Fields] OR "depressives" [All Fields])

AND ("Radiology"[Mesh] OR “Imaging Genomics”[All Fields] OR "Nuclear Medicine"[All Fields] OR "Radiation Genomics"[All Fields] OR "Radiation Oncology"[All Fields] OR "Radiology, Interventional"[All Fields] )

**Table S1.** Changes in the number of publication types from 2002 to 2021

| **Publication Type** | **2002-2006** | | **2007-2011** | **2012-2016** | | **2017-2021** | **total** |
| --- | --- | --- | --- | --- | --- | --- | --- |
| **Case Reports** | | 134 | 156 | 167 | 211 | | 668 |
| **Review** | | 47 | 60 | 106 | 182 | | 395 |
| **Comparative Study** | | 100 | 98 | 97 | 65 | | 360 |
| **Randomized Controlled Trial** | | 19 | 23 | 75 | 110 | | 227 |
| **Multicenter Study** | | 16 | 18 | 62 | 89 | | 185 |
| **Clinical Trial and Study** | | 42 | 21 | 43 | 61 | | 167 |
| **Meta-Analysis** | | 2 | 3 | 23 | 46 | | 74 |
| **Systematic Review** | | 0 | 1 | 16 | 40 | | 57 |

**Table S2.** Top 10 most citation for included publications.

| **Reference title** | **DOI** | **Article title** | **Inner citations** | **Total citations** |
| --- | --- | --- | --- | --- |
| J Neurol Neurosurg Psychiatry. 1960 Feb;23:56-62 | <https://jnnp.bmj.com/content/23/1/56> | A rating scale for depression | 181 | 25656 |
| Neuroimage. 2002 Jan;15(1):273-89 | <https://doi.org/10.1006/nimg.2001.0978> | Automated anatomical labeling of activations in SPM using a macroscopic anatomical parcellation of the MNI MRI single-subject brain | 109 | 11031 |
| Acta Psychiatr Scand. 1983 Jun;67(6):361-70 | <https://doi.org/10.1111/j.1600-0447.1983.tb09716.x> | The hospital anxiety and depression scale | 105 | 29387 |
| J Psychiatr Res. 1975 Nov;12(3):189-98 | <https://doi.org/10.1016/0022-3956(75)90026-6> | "Mini-mental state". A practical method for grading the cognitive state of patients for the clinician | 104 | 64605 |
| Arch Gen Psychiatry. 1961 Jun;4:561-71 | <https://doi.org/10.1001/archpsyc.1961.01710120031004> | An inventory for measuring depression | 84 | 26204 |
| Biol Psychiatry. 2007 Sep 1;62(5):429-37 | <https://doi.org/10.1016/j.biopsych.2006.09.020> | Resting-state functional connectivity in major depression: abnormally increased contributions from subgenual cingulate cortex and thalamus | 84 | 1505 |
| Br J Psychiatry. 1979 Apr;134:382-9 | <https://doi.org/10.1192/bjp.134.4.382> | A new depression scale designed to be sensitive to change | 78 | 9929 |
| JAMA Psychiatry. 2015 Jun;72(6):603-11 | <https://doi.org/10.1001/jamapsychiatry.2015.0071> | Large-Scale Network Dysfunction in Major Depressive Disorder: A Meta-analysis of Resting-State Functional Connectivity | 76 | 940 |
| Br J Soc Clin Psychol. 1967 Dec;6(4):278-96 | <https://doi.org/10.1111/j.2044-8260.1967.tb00530.x> | Development of a rating scale for primary depressive illness | 72 | 244 |
| Neuroimage. 2006 Jul 1;31(3):968-80 | <https://doi.org/10.1016/j.neuroimage.2006.01.021> | An automated labeling system for subdividing the human cerebral cortex on MRI scans into gyral based regions of interest | 68 | 6625 |


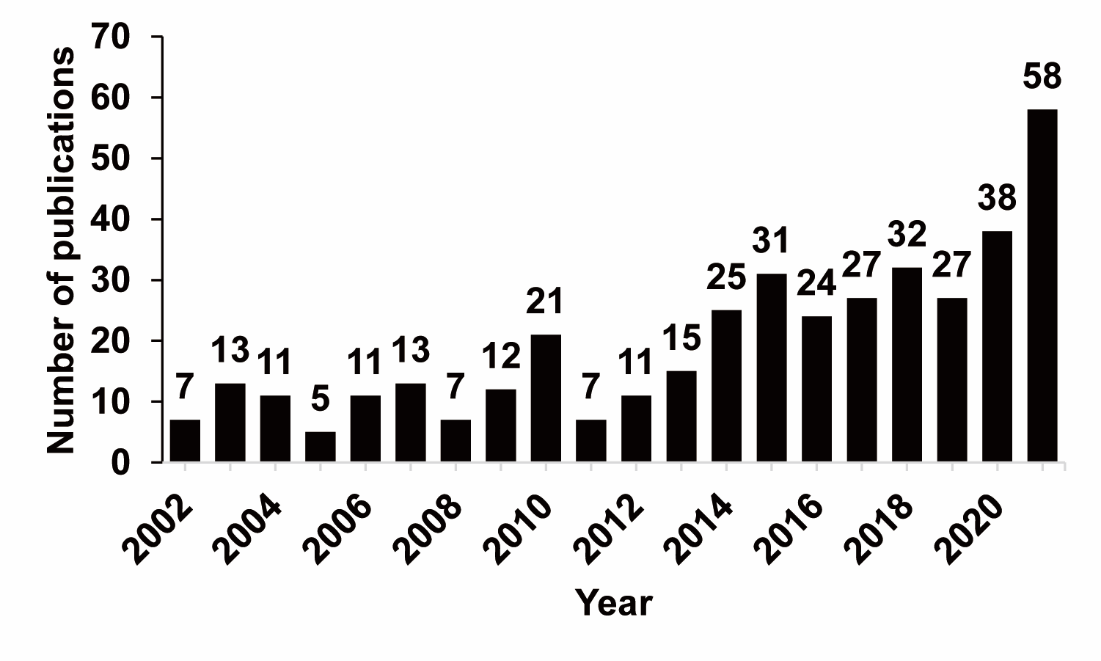


**Figure S1.** Number of publications of Review in MDD and radiology studies

**Supplemental information 2** Code of Latent Dirichlet allocation methods

from ast import Try

from nltk.tokenize import RegexpTokenizer

#from stop_words import get_stop_words

from nltk.stem.porter import PorterStemmer

from sklearn.utils import shuffle

from gensim import corpora, models

import pandas as pd

import logging

import pickle

import numpy as np

import os,sys

from matplotlib import pyplot as plt

logging.basicConfig(level = logging.INFO,format = '%(asctime)s - %(name)s - %(levelname)s - %(message)s')

logger = logging.getLogger(__name__)

from gensim.models.ldamulticore import LdaMulticore

def dumppick(filepath,Year):

corpus = []

tokens = []

df = pd.read_csv(filepath,sep='\t',encoding="utf-8-sig",error_bad_lines=False)

df = df[df["Abstract"].isna()!=True]

df.astype({'year': 'int32'})

df = df[df.year<Year]

for line in df["Abstract"]:

corpus.append(line.strip())

del df

en_stop = ['i', 'me', 'my', 'myself', 'we', 'our', 'ours', 'ourselves', 'you', 'your', 'yours', 'yourself', 'yourselves', 'he', 'him', 'his', 'himself', 'she', 'her', 'hers', 'herself', 'it', 'its', 'itself', 'they', 'them', 'their', 'theirs', 'themselves', 'what', 'which', 'who', 'whom', 'this', 'that', 'these', 'those', 'am', 'is', 'are', 'was', 'were', 'be', 'been', 'being', 'have', 'has', 'had', 'having', 'do', 'does', 'did', 'doing', 'a', 'an', 'the', 'and', 'but', 'if', 'or', 'because', 'as', 'until', 'while', 'of', 'at', 'by', 'for', 'with', 'about', 'against', 'between', 'into', 'through', 'during', 'before', 'after', 'above', 'below', 'to', 'from', 'up', 'down', 'in', 'out', 'on', 'off', 'over', 'under', 'again', 'further', 'then', 'once', 'here', 'there', 'when', 'where', 'why', 'how', 'all', 'any', 'both', 'each', 'few', 'more', 'most', 'other', 'some', 'such', 'no', 'nor', 'not', 'only', 'own', 'same', 'so', 'than', 'too', 'very', 's', 't', 'can', 'will', 'just', 'don', 'should', 'now', 'd', 'll', 'm', 'o', 're', 've', 'y', 'ain', 'aren', 'couldn', 'didn', 'doesn', 'hadn', 'hasn', 'haven', 'isn', 'ma', 'mightn', 'mustn', 'needn', 'shan', 'shouldn', 'wasn', 'weren', 'won', 'wouldn']

p_stemmer = PorterStemmer()

logging.info("wenbenyuchuli")

tokenizer = RegexpTokenizer(r'[A-Za-z]+')

for i,text in enumerate(corpus):

if i%1000==0:

logging.info(f"{i} line done")

raw = text.lower()

token = tokenizer.tokenize(raw)

stop_remove_token = [word for word in token if (word not in en_stop and len(word)>1)]

stem_token = [p_stemmer.stem(word) for word in stop_remove_token]

tokens.append(stem_token)

# tokens.append(token)

# print tokens

logging.info("start")

logging.info("basic")

dictionary = corpora.Dictionary(tokens)

logging.info("word bags")

texts = [dictionary.doc2bow(text) for text in tokens]

logging.info("finished")

logging.info("start tfidf")

texts_tf_idf = models.TfidfModel(texts)[texts]

pickle.dump(texts, open("text_dtm.pickle","wb"))

pickle.dump(texts_tf_idf, open("texts_tf_idf_dtm.pickle","wb"))

pickle.dump(dictionary, open("dictionary.pickle","wb"))

def loadpcik():

texts = pickle.load(open("text_dtm.pickle","rb"))

texts_tf_idf = pickle.load(open("texts_tf_idf_dtm.pickle","rb"))

dictionary = pickle.load(open("dictionary.pickle","rb"))

return texts, texts_tf_idf,dictionary

#dumppick()

def calc_n_of_lda(filename,Year,start,end):

dumppick(filename,Year)

texts, texts_tf_idf, dictionary = loadpcik()

"""

print("**************LSI*************")

lsi = models.lsimodel.LsiModel(corpus=texts, id2word=dictionary, num_topics=20)

texts_lsi = lsi[texts_tf_idf]

print(lsi.print_topics(num_topics=20, num_words=10))

"""

logging.info("**************LDA*************")

ppl = []

for num_topics in range(start,end,1):

texts = shuffle(texts)

lda = LdaMulticore(corpus=texts,iterations=50, id2word=dictionary, num_topics=num_topics,passes=10,per_word_topics=True)

#texts_lda = lda[texts_tf_idf]

# print(lda.print_topics(num_topics=num_topics, num_words=10),file =out)

# ppl.append(np.exp2(-lda.log_perplexity(texts))

ppl.append(lda.log_perplexity(texts))

plt.plot( range(10,60,1),ppl)

plt.title("num_topics(x) - perplexity(y)")

plt.savefig("prop.png")

plt.show()

return lda, texts, texts_tf_idf, dictionary, ppl

def load_lda(filename,num_topics):

texts, texts_tf_idf, dictionary = loadpcik()

lda = LdaMulticore(corpus=texts,iterations=100, id2word=dictionary, num_topics=num_topics,passes=20,per_word_topics=True)

lda.save("./ldamd/{}tpc+{}".format(num_topics,filename[9:18]))

return lda, texts, texts_tf_idf, dictionary,

def saveldatpcw(lda,num_topics):

tpcn = num_topics

tpcw = pd.DataFrame(columns=[i for i in range(1,11)])

for i in range(tpcn):

tpcw.loc[i] = [ w for w,p in lda.show_topic(i)]

tpcw.to_csv("./newdata/tpcw.csv")

def get_cite_n_dmt(dictionary,citenum=0,):

citenum=0

corpus = []

tokens = []

df = pd.read_csv("pubmed_result_parsed.csv",sep=',',encoding="utf-8-sig")

df = df[df["cite"]==citenum]

df = df[df["Abstract"].isna()!=True]

for line in df["Abstract"]:

corpus.append(line.strip())

del df

en_stop = [ str(i).strip() for i in open("stopwords.txt",encoding="utf-8-sig") ]

p_stemmer = PorterStemmer()

logging.info("wenbenyuchuli")

tokenizer = RegexpTokenizer(r'[A-Za-z]+')

for i,text in enumerate(corpus):

if i%1000==0:

print(i)

raw = text.lower()

token = tokenizer.tokenize(raw)

stop_remove_token = [word for word in token if (word not in en_stop and len(word)>1)]

stem_token = [p_stemmer.stem(word) for word in stop_remove_token]

tokens.append(stop_remove_token)

texts_cite_n = [dictionary.doc2bow(text) for text in tokens]

return texts_cite_n

def getallcited2tpc(lda,texts,filename,Year):

tpc1 = []

tpc2 = []

for i in texts:

tpc = lda.get_document_topics(i)

tpc = sorted(tpc,key=lambda x:-x[1])

tpc1.append(tpc[0][0])

if len(tpc)>1:

tpc2.append(tpc[1][0])

else:

tpc2.append(lda.num_topics+1)

df = pd.read_csv(filename,sep='\t',encoding="utf-8-sig")

df = df[df["Abstract"].isna()!=True]

df = df[df.year<Year]

df["tpc1"] = tpc1

df["tpc2"] = tpc2

df.to_csv(filename.replace(".csv","_with_topic.csv"),sep='\t',encoding="utf-8-sig")

return tpc1,tpc2

def grap(tpc1,tpc2,tpcn,filename):

from collections import Counter

CC = Counter(tpc1)

import networkx as nx

G = nx.Graph()

for i in range(tpcn):

G.add_node(i,num=CC[i])

#edgscount = Counter([(i,j) for i,j in zip(tpc1,tpc2)])

#for edgs,count in edgscount.items()

edgeslist = [(i,j) for i,j in zip(tpc1,tpc2) if j<tpcn]

G.add_edges_from(edgeslist,Weight=0)

for i,j in zip(tpc1,tpc2):

if j >= tpcn:

continue

G.edges[i,j]["Weight"]+=1

nx.write_graphml(G,filename.replace(".csv",".graphml"),encoding="utf8")

return G

def Grap_Add_tpcname():

import pandas as pd

c = pd.read_excel("glioblastoma\\topics-gbm.xlsx")

pic = {tpcid:name for tpcid,name in zip( range(50),c["topics"])}

import networkx as nx

g = nx.read_graphml("glioblastoma\\Glioblastoma_50.graphml")

for i in range(50):

g.node[str(i)]["name"] = pic[i]

nx.write_graphml(g,"glioblastoma\\Glioblastoma_50.graphml_addname.graphml",encoding="utf8")

def main(Year):

filename = "total.csv"

if not os.path.exists("./newdata"):

os.mkdir("./newdata")

if not os.path.exists("./ldamd"):

os.mkdir("./ldamd")

start, end = map(int, str(input("Enter calculation subject range comma-separated (example 10,60): ")).split(","))

calc_n_of_lda(filename,Year, start, end)

num_topics = int(input("Enter the number of topics："))

lda, texts, texts_tf_idf, dictionary = load_lda(filename,num_topics)

saveldatpcw(lda,num_topics)

logging.info("LDA finish")

logging.info("in each publication")

tpc1,tpc2 = getallcited2tpc(lda,texts,filename,Year)

logging.info("fig formation")

grap(tpc1,tpc2,num_topics,filename)

if __name__ == "__main__":

Year = 2022

main(Year)
